# “Every day is a battle against Parkinson’s”—a qualitative study on older adults at the long-term care facilities in Chennai

**DOI:** 10.3389/fpubh.2026.1747471

**Published:** 2026-02-11

**Authors:** Rajyasree A., J. Balamurugan

**Affiliations:** Department of Social Sciences, School of Social Sciences and Languages, Vellore Institute of Technology, Vellore, Tamil Nadu, India

**Keywords:** India, institutionalized older adult, long-term care facilities, older adults, Parkinson’s disease, qualitative research, thematic analysis

## Abstract

**Background:**

Parkinson’s disease is the second most common progressive neurodegenerative disorder affecting both motor and non-motor functions, with men showing higher susceptibility. It comprises unilateral and axial symptoms leading to severe functional disability. Also, there are numerous unexplored symptoms, since Parkinson’s is called “Snowflake Disease.”

**Objective:**

The objective of the study is to explore and understand life after Parkinson’s disease in older adults living in long-term care facilities.

**Methodology:**

A descriptive qualitative study utilizing purposive sampling was conducted among 22 older adults with Parkinson’s disease (PD) aged 60 to 89 years (±74.5). In-depth interviews were conducted, with six older adults excluded due to fatigue and cognitive problems. The final sample of 16 interviews achieved data saturation and is potentially valid for analysis.

**Results:**

A thematic analysis was conducted, resulting in the identification of six principal themes: (1) Identity and Self-perception, (2) Emotional Dysphoria, (3) Neurofunctional Impact, (4) Acclimatization to Therapy, (5) Need for Support Network, and (6) Existential Reflection. The findings illustrate the crucial importance of perceived autonomy and family support in the process of attaining psychosocial well-being.

**Conclusion:**

It indicates the interplay of factors influencing older adults with Parkinson’s disease in long-term care facilities. It also underscores the crucial role of healthcare professionals, policymakers, and caregivers in designing tailored strategies for multifaceted challenges associated with Parkinson’s disease.

## 1 Introduction

Parkinson’s disease is the second most common neurodegenerative disease among older adults, showing a 21.7% increase in prevalence over the two decades (1). As per the Indian Council of Medical Research (ICMR), Parkinson’s leads to 1.8% of the total disease-adjusted life years (1, 2). It is a progressive disease characterized by motor symptoms like tremors, rigidity, and slow movement (bradykinesia), as well as non-motor symptoms like mood disorders, sleep, and memory problems (3, 4). Research shows that there is an 80% rate of dementia among older adults suffering from Parkinson’s disease (5–7). The well-being depends on socioeconomic determinants like work status and independence, alongside health factors including comorbidities, mental health, and quality of life (8–10). While Parkinson’s disease has no cure, diet, exercise, and treatment can help manage daily functioning and critical symptoms (11–13).

The pathophysiological burden of Parkinson’s disease is significantly deepened when non-motor symptoms coexist with comorbidities (14–16). Non-motor symptoms represent a substantial dimension of disease burden that frequently exceeds motor symptoms in affecting older adults’ quality of life and psychological well-being within institutional settings (17). These manifestations encompass neuropsychiatric disorders (depression, anxiety, apathy), cognitive impairment, sleep disturbances, and before ‘and emotional processing difficulties (18, 19). For older adults in long-term care facilities, these symptoms directly influence how individuals navigate daily life, maintain relationships, and experience autonomy and dignity (20). A complete understanding of non-motor symptoms accounts for both the underlying mechanisms and the institutional outcomes that follow.

Affective disturbances constitute the most prevalent non-motor manifestations in PD (21). Depression affects 35–50% of patients and correlates with reduced quality of life independent of motor disability. This is a reflection of dysregulation in monoaminergic systems that goes beyond the typical dopamine deficit (22), while anxiety disorders (20–40% of patients) and apathy (up to 40%) collectively reduce motivation for social participation and engagement (23). These affective difficulties are not only because of psychological origin but also of neurobiological changes that impact serotonergic, noradrenergic, and GABAergic systems (24). These are compounded by loss of independence within institutional contexts (25).

Beyond affective disorders, alterations in social cognition and emotion processing substantially affect relational life in care settings (25, 26). Patients exhibit difficulties recognizing emotional expressions and interpreting social cues, with neurobiological changes that have their roots in affecting the functioning of the amygdala and the prefrontal cortex (27). The social-cognitive deficits undermine the development and maintenance of significant relationships that are crucial for psychological well-being.

Alexithymia is a difficulty in identifying and describing emotions, which affects the quality of life in Parkinson’s patients (28). The systematic evidence regarding alexithymia in PD has shown significant negative implications for quality of life (29), creating barriers for institutionalized older adults to articulate emotional needs, straining interpersonal relationships and deepening isolation (30).

Sleep disturbances, particularly REM sleep behavior disorder, represent a critical non-motor manifestation with wide-ranging neuropsychological complications (31, 32). RBD disrupts nocturnal rest and is associated with accelerated cognitive decline and behavioral changes in older adults with PD (33). Recent evidence from systematic reviews has shown the two-way relationship between REM sleep behavior disorder and cognitive function in older adults with PD, confirming RBD as a predictor of the severity and course of cognitive decline (33). Within institutional settings, sleep dysfunction cascades into daytime cognitive impairment, diminishing residents’ capacity for meaningful engagement and decision-making (34). This sleep–cognition specifies a mechanistic path through which non-motor symptoms affect the psychological capacity for adaptation and well-being.

The non-motor symptoms: affective disturbances, social-cognitive changes, alexithymia, and sleep dysfunction are interrelated. These constitute integral neurobiological features of PD’s pathophysiology that fundamentally shape subjective experience (35). Unlike motor limitations, non-motor manifestations compromise psychological needs as articulated within Maslow’s hierarchy: belongingness through disrupted social cognition and isolation, esteem through apathy, and self-actualization through cognitive constraints and emotional dysregulation (36, 37). This mechanistic apprehension is crucial for comprehending how PD creates psychosocial risks in an institutional setting that are beyond the occurrences in community-dwelling older adults.

Despite growing recognition of these challenges, a critical gap exists in comprehending the experiences of older adults with PD in long-term care facilities in India. Quantitative studies in India have reported the incidence of symptoms, measured quality of life indicators, and compared functional impairments across PD (38, 39). However, these methods cannot capture the subjective perception and response to the disease. The shift to long-term care facilities affects fundamental human needs for social connection and meaningful relationships (40). Older adults with Parkinson’s disease in institutions often experience decreased social interaction, weakened family bonds, and difficulty maintaining relationships (41). Crucially, these relational deficits emerge alongside non-motor neuropsychiatric symptoms. Notably, changes in social cognition and alexithymia actively reduce the potential for interpersonal interactions, thus increasing vulnerability.

The theoretical framework that underlies this investigation is an integration of Maslow’s Hierarchy of Needs and the Biopsychosocial Model of Illness. Maslow’s hierarchy of needs identifies love and belonging as essential human requirements (42, 43). While institutional settings may adequately address physiological and safety needs (44, 45), Higher-level needs for belongingness, esteem, and self-actualization are threatened when older adults with PD feel forced into care environments that conflict with their personal or cultural preferences (46). Qualitative research suggests that older adults with PD in care settings may experience abandonment and isolation (47–49). Particularly in South Indian contexts, support by the family and expectation for care are high when compared with Western countries. The study of the experiences of older adults with PD in long-term care has remained an unexplored area, especially in the South Indian context, where there are culturally rooted notions of family involvement that differ significantly from the paradigms of institutional care (50).

The descriptive qualitative study addresses the significant gap by using Maslow’s Hierarchy of Needs and the biopsychosocial model as guiding frameworks. It explores the experiences of older people living with Parkinson’s disease in long-term care facilities in Chennai, Southern India, with a special focus on the psychological, emotional, relational, and existential aspects of institutional life as influenced by non-motor symptoms and cultural factors.

## 2 Materials and methods

### 2.1 Study design

The first author (RA), a doctoral candidate in psychology, and the corresponding author (BJ) have completed PhD., M.A can be replaced with PhD., M.A in Sociology. Our interest stems from recognizing gaps in understanding the psychosocial experiences of older people with chronic conditions in long-term care facilities. It further proceeded by narrowing down to the Parkinson’s population. We realize that our interdisciplinary, cultural, and experiential positions (including both academic and work experiences) may affect the interpretation of the participant quotes. Hence, we have used several strategies to ensure that the findings reflect participants’ realities, while recognizing that interpretation forms an intrinsic part of qualitative investigation. The combination of these methods enhances trustworthiness and transparency in reporting perspectives. The researchers also conducted peer debriefing meetings during the pre-data collection, data collection, and post-analysis phases bi-weekly. These sessions examined the emerging codes, themes, and interpretations, allowing for recognition of alternate explanations and findings to be based on the interviews rather than assumptions or biases that researchers held. Before the initiation of data collection, the research team involved in bracketing activities through consciously acknowledging and recording their assumptions regarding institutional care, chronic affliction, Parkinson’s disease, and family dynamics, particularly in the South Indian context. Field notes recorded observations and thoughts during the process of data collection, allowing for a process of ongoing reflection. No previous relationships with older adults with PD or institutions related to them existed for the researchers that would have effectively nullified role conflict and bias. The combination of these methods, involving peer debriefing, bracketing, field notes, and audit trails, together ensures findings reflect realities among older adults with PD while recognizing interpretation as an inherent component of qualitative study.

The current study employed Thorne’s (2016) interpretive description method, which is designed to produce a pragmatic description of health phenomena while maintaining rigor and integrity in methodology (51). Sally Thorne’s method was appropriate for studying the everyday battles of older adults with PD due to its systematic approach to analyzing individual narratives and comparative patterns, for informing improved clinical practice and care delivery.

Qualitative data were obtained through contextual exploration using 22 individual descriptive narrative interviews. The rationale for this approach is that it provides a rigorous method for the data acquisition process through in-depth interviews. Observations of the care environment and practices, physiological conditions, pedagogical shifts, cultural nuances, patterns of medical checkups, emotional support, and care capture the essence of the mourning of research older adults with Parkinson’s disease. This descriptive qualitative study on older adults with Parkinson’s disease was performed in October 2024 and November 2024.

### 2.2 Sample recruitment and data exclusion

The interviewees with several comorbidities (as mentioned in Table 1) and at various stages (stages 1 to 4), as mentioned in the inclusion criteria, were selected. A total of 5 long-term care facilities were contacted: 1 high-end care in the south region and 4 basic care facilities in the north region of Chennai. 22 older adults with Parkinson’s disease were initially invited to take part in the study. 6 were excluded from the data analysis to maintain data integrity and achieve meaningful analysis. Exclusions were due to: (1) incomplete interviews as a consequence of profound motor symptoms limiting proper interaction (*n* = 2), (2) participants withdrawn their consent during an ongoing interview due to uneasiness and fatigue (*n* = 2), and (3) failure to finish interviews due to the development of cognitive impairment during data gathering, affecting both the narration and response quality (*n* = 2).

**Table 1:** Reflects the socio-demographic details and clinical profile (*n* = 16).

Variables	*n* (%)
Age	
60–69	3 (18.75)
70–79	8 (50.0)
80–89	5 (31.25)
Gender	
Male	11 (68.75)
Female	5 (31.25)
Educational status	
No formal education	1 (6.25)
Primary	3 (18.75)
High school	2 (12.5)
Higher secondary	4 (25.0)
Undergraduate & above	6 (37.5)
Area of residence	
Urban	13 (81.25)
Rural	3 (18.75)
Marital status	
Unmarried	1 (6.25)
Married	4 (25.0)
Separated	3 (18.75)
Divorced	2 (12.5)
Widow	5 (31.25)
Widower	(6.25)
Existing comorbidities	
Lung disease	7 (43.75)
Asthma	1 (6.25)
Diabetes	8 (50.0)
Back pain	5 (31.25)
Heart disease	4 (25.0)
Nervous disorder	1 (6.25)
Blood pressure	6 (37.5)
Skin disease	3 (18.75)
Paralysis	2 (12.5)
Tuberculosis	1 (6.25)
Rheumatism/Arthritis	2 (12.5)
Kidney disease	1 (6.25)
Neurocognitive and sensory motor problems	
Memory	2 (12.5)
Vision	7 (43.75)
Speech	5 (31.25)
Walking	11 (68.75)
Hearing	1 (6.25)
Type of treatment	
Deep brain stimulation	1 (6.25)
Medicines	15 (93.75)
Physiotherapy	9 (56.25)
Period of diagnosis	
0.6 Months to 1 year	5 (31.25)
1.1–2 years	3 (18.75)
2.1–3 years	2 (12.5)
3.1–4 years	6 (37.5)
Frequency of hospital visits	
Rarely	9 (56.25)
Once a week	4 (25.0)
Twice a week	2 (12.5)
Frequently	1 (6.25)

The final sample was 16 older adults with Parkinson’s disease from the 5 long-term care facilities. The gender distribution was 11 men and 5 women, which naturally aligned with the epidemiology of Parkinson’s disease, showing higher male prevalence. Inclusion criteria comprised older adults with PD of all genders, aged 60 years and above, stages (0.5 to 4.0), with disease duration exceeding six months and above. Exclusion criteria encompassed stage V older adults with Parkinson’s disease (considering the disease progression, as per the Hoehn and Yahr stages for Parkinson’s disease), those are some of whom are bedridden and have cognitive, speech, and extreme non-motor complications, and those within 6 months of diagnosis (52). In addition, stage V older persons with PD who demonstrated high levels of physical or cognitive dependency were also excluded from the study. Those are more likely to face challenges in care, unmet needs, and functional limitations, and this may influence their perceptions of care quality. Thus, the exclusion may have influenced the satisfaction scores, which may result in the lack of representation of limited favorable care experiences, and potentially amplify positive evaluations. The results are, therefore, likely to represent the experiences of older adults with PD who are capable of participating in the study (Table 2).

**Table 2:** Sample questions in the interview schedule.

S. No	Questions
1.	Describe the experiences before, during, and after being diagnosed with Parkinson’s disease.
2.	How did the physiological and non-motor symptoms affect you? In what ways?
3.	Do you ever feel hopeless or isolated after the diagnosis of PD? Could you please explain more about that?

### 2.3 Data saturation

Data saturation was reached by corroborating and cross-verifying the themes of 16 interviews, which revealed repeated patterns. The preliminary analysis was initiated after 12 interviews, and saturation was attained by interview 14. Also, no new experiences or additional themes were arising from the last 3 interviews. Another 2 interviews were conducted to verify saturation and extensive data collection. Determining the point of saturation was done simultaneously with ongoing analysis during the data collection process. The researchers observed thematic and semantic richness across the 6 identified themes. This sample size is consistent with desirable qualitative research guidelines, which suggest 12 to 20 participants are generally adequate to achieve saturation in phenomenological investigations (53). Participant diversity based on stages of Parkinson’s disease (1–4), age (60–89 years), and long-term care facilities also reflected the sample sufficiency in representing the wide range of older adults with Parkinson’s disease in the socio-cultural context.

### 2.4 Data collection

The sample population for this study was obtained using purposive sampling based on the predetermined criteria mentioned in (2.2). The overall population comprises all genders with varied socio-economic and cultural backgrounds. This study employs descriptive narrative interviews conducted among 22 older adults with Parkinson’s disease, out of which 16 are considered potential interviewees for the qualitative thematic analysis. The older adults with PD and their caregivers were given information sheets and consent forms, with explanations given verbally. For participants who have difficulties in comprehension, validated caregiver consent forms were used, and consent was sought from the legal guardians. No audio-visual recordings were done. Participants were intimated that they have the right to withdraw at any time. An initial rapport was built at this stage to gain immersive trust to share their journey with PD. The observations of the long-term care facilities (as listed in section 2.1) were noted down separately. At the first instance, the health- and demographic-related information was collected. A unique pseudonym was given to each study participant, and the data were encrypted with that specific pseudonym, ensuring confidentiality. The semi-structured schedule of interviews and notes on research documentation were used, in which the in-depth interviews were audio-recorded. The researcher probed the older adults with PD to describe more about the interpersonal perceptions toward the disease, experiences, and attitudes regarding the diagnosis of PD, continued by the treatment, symptomatic management, and coping with Parkinson’s disease. The sample questions for the interview has been listed in the Table 2. To understand the scenario comprehensively, the interviewer continued with questions such as “Could you please elaborate more on this?” The period of interviews takes place between 40 and 60 min. The interviewer concluded the session by asking, “Is there anything you would like to share regarding this journey with Parkinson’s disease?”

### 2.5 Data analysis

The semi-structured interview schedule was initially prepared by the primary researcher in English and translated into Tamil to facilitate participant comfort and authentic expression. It had a set of 14 questions the all interviews were conducted in Tamil by the first author (RA), who is fluent in both Tamil and English, given the older adults with Parkinson’s disease’s linguistic context and preference for their native language. The first author transcribed audio-recorded interviews verbatim in Tamil immediately after each interview, ensuring the preservation of nuances, colloquialisms, and emotional expressions. Concurrently, discussions were held with the second author (BJ) to gather the requisite insights. The Tamil transcripts were then translated into English. English transcripts formed the basis for coding, analysis, and reporting work. A back-translation process was used to ensure the quality of translations and preserve the original meaning: a bilingual expert (not involved in this study) translated selective English transcripts back into Tamil, and they could be compared with the original Tamil transcripts to identify discrepancies. The researchers’ expertise in both languages and their knowledge of the South Indian culture allowed them to understand idiomatic expressions and cultural nuances.

Researchers engaged in several readings of the transcripts to achieve an extensive understanding. Preliminary insights and constructive discrepancies were recorded for further disputes. Two researchers (RA, BJ) independently coded 20% of the transcripts and discussed the coding choices. This ensured uniformity of coding before the primary researcher completed the remaining analysis. Codes were generated in English from the translated transcripts and organized. For both Tamil transcription and English translation, we were using MS Excel and MS Word. The thematic coding and analysis were also conducted by using the same approach to arrange codes with relevant quotations and rationale for ease of data audit by the authors. Code generation was done inductively by monitoring word-by-word, line-by-line, excluding the predetermined assumptions about the psychosocial struggles of older adults with PD. Each meaningful part of the text was given code. To avoid repetitions and unnecessary eliminations, the obtained codes were reviewed and organized meticulously. Again, both authors reviewed the coded output to verify coding consistency and analytical logic. However, when ambiguity arose during coding, the research team referred back to both the Tamil transcripts and English translations to verify meaning and context. This bilingual verification ensured that the conceptual essence of the narratives was accurately captured in the English codes. Codes were sub-categorized into specific themes according to the representation of data acquired. The iterative process of finalizing the codes and themes persisted until it exactly resembled the meaning of the shared journey of older adults with PD. The finalized codes, subthemes, and themes are represented in a tabulated form.

### 2.6 Rigor

Trustworthiness was achieved by focusing on four key elements: credibility, transferability, dependability, and confirmability (54). Credibility is confirmed by the triangulation method by involving various data sources, like observations, documentation, member checking and peer debriefing between the two researchers. The dual-researcher coding approach (RA and BJ) provided investigator triangulation, reducing individual bias in interpretation. In order to establish transferability, the demographic details of older adults with PD and their long-term care facilities are explained. This study adheres to the checklist of Braun and Clarke’s thematic analysis (55). Alongside, the study accompanies the COREQ (Consolidated Criteria for Reporting Qualitative Research) guidelines. It also acts as an authorized standard (EQUATOR network) for reporting qualitative studies for analytical rigor. Out of 32 items, 29 items were satisfied, contributing to 90.6% of the overall COREQ checklist. An audit trail was maintained with methodological decisions, like the construction of the coding framework, reflexive accounts of self-positioning by the researcher, and documentation of consensus discussions between RA and BJ, to ensure dependability. Confirmability was enhanced by maintaining objectivity and documenting the researcher’s experience, both before and during the session. This bilingual process for back translation and verification maintained the true voices and cultural expressions of older adults with PD with analytical rigor and procedural rigor.

### 2.7 Ethics approval

The study was approved by the Institutional Ethical Committee for Human Studies (IECH) at Vellore Institute of Technology, under the (VIT/IECH/2024/15 IECH/ 20 April 2024/16). All procedures were conducted in accordance with the Declaration of Helsinki and the Health Insurance Portability and Accountability Act (HIPAA, 1996). Informed consent was sought from all participants and/or their guardians before data collection.

## 3 Results

Participants were older adults of South Indian descent residing in long-term care settings in Chennai, within the 60 to 89 age range, with an average age of 74.5. However, the majority are male, as referred to in section 2.2. Demographic factors were used to interpret the themes that emerged from the analysis. The older adults with Parkinson’s disease (PD) had presented diverse marital statuses, ranging from married, widowed, to unmarried, as indicated in Table 1, which follows the format used by Chauhan (2025) (56). Also, it unveils comorbidity types, the duration of movement disability, and pre-morbidity characteristics. The researchers performed the thematic analysis and arrived at six significant themes presented in Table 3. Those are: (1) Identity and Self-perception, (2) Emotional Dysphoria, (3) Neurofunctional Impact, (4) Acclimatization to Therapy, (5) Need for support network, and (6) Existential reflection. The 106 codes are generated and consolidated into 81 subthemes, which are integrated into the 6 principal themes.

**Table 3:** It indicates the themes and sub-themes (*n* = 16).

S. No	Themes	Sub-themes
1	Identity and self-perception	Coping with the diagnosis of PD
		Adaptation to lifestyle changes
		Resilience enhancement and treatment acceptance
2	Emotional dysphoria	PD-related depression and persistent sadness
		Anticipatory fear and anxiety
		Resilience and coping strategies
3	Neurofunctional impact	Chronic physical limitations and mobility complications
		Cognitive dysfunction and executive challenges
		Financial constraints and resource management
4	Acclimatization to therapy	Experiences and adaptation with therapy
		Medication and side effects of PD
		Contribution and support of healthcare providers
5	Need for a support network	Interpersonal relationships changes
		Social Isolation and community withdrawal
		Peer support and mutual connection
6	Existential reflection	Spiritual coping mechanisms and faith building
		Gratitude expression and meaning making
		Reflections on mortality

### 3.1 Identity and self-perception

This theme explains how older people with PD construct their self-concept in relation to the diagnosis. They have reported experiences of being seen as differently abled within their social contexts. The participants reported feelings of being overwhelmed and emotionally distressed after the diagnosis of PD (P16). Initially, older adults with PD had wondered about the way to react to the situation, where they had recently been diagnosed with Parkinson’s disease. Hearing from the neurologist/neurosurgeon that this was a rare disease and there was no cure for this neurodegenerative disorder had led them to a state of denial; they had refused to believe that they had been diagnosed with a disease that had no cure, till death (P12). They have shared mixed feelings regarding conquering the reasons for the occurrence of the chronic condition and gaining insights into the symptoms, frequency of symptoms, severity, and development of the movement disability (P4). Some older adults with PD had questioned themselves as to how they had even arrived at a disease where they were still active and energetic (P6). Those narratives of older adults with Parkinson’s disease were distressed that, from the initial days, efforts to be healthy had been taken, and the individual had thus arrived at the disease (P7). Attempts to continue living despite the diagnosis of PD were mentioned. The perception of people around them had also affected them since it led to the fear of judgments and speculations. They have expressed feelings of embarrassment when they took part in public gatherings, especially in a social or new environment. They also described Guilt and lowered self-esteem after PD diagnosis.

“When the neurologist told me ‘You have Parkinson’s,’ “I did not know what to do” (P16).

“I was shocked when the physician informed me that I had Parkinson’s. I did not believe it during the early months after the diagnosis” (P12).

“When I was first diagnosed, I had trouble believing it. I could not accept it.” I would wonder, ‘Why me?” (P4).

“It was hard to understand that someone as active as I could develop Parkinson’s disease” (P6).

“I had worked my whole life, and to be told that I had Parkinson’s was a betrayal of my body” (P7).

“When I was told that I was diagnosed with Parkinson’s, I blanked out. All I could think about was how this would change everything. I reminded myself every day that life was still worth living, even on the bad days” (P2).

### 3.2 Emotional dysphoria

The emotional dysphoria theme captures the immediate emotional response of older adults with PD who have experienced the disease symptoms and functional limitations. Unlike the reflective meaning-making process described in Theme 6 (Existential Reflection), these emotions represent present-focused distress triggered by specific symptoms and daily challenges. The fear of falling, public judgment and acceptance, feelings of helplessness due to the progression of the disease and the lack of a cure were elaborated by older adults with PD. These feelings are associated with tremors, medication side effects, and loss of independence. They also described the fear of falling or loss of balance as traumatic (P11). This was reflected in the narratives as older adults with Parkinson’s disease shared their experiences and forthcoming hurdles after the diagnosis (P5). One of the older adults with Parkinson’s disease felt that their existence is no longer beneficial for their children (P3).

“The everyday activities were a task, needing assistance and being dependent on carers weakened me so much and made me so vulnerable. It feels like I’m a soldier going to war on a battlefield” (P11).

“I used to enjoy gardening; now, I’m not able to hold a spade properly. What would happen if I fell and nobody was there to lift me? Sometimes, at particular times, I’d hope that everything just stops.” The struggle is so exhausting” (P5).

“I feel that I’m a burden to my kids. But that hug from my granddaughter makes this experience justify itself. The tremors and stiffness put me in fear that I’ll inadvertently drop objects in front of other people.” I do not know how I will get through this” (P3).

### 3.3 Neurofunctional impact

This theme presents both physical symptoms and perceived changes in mental health. The older adults outlined the consequences of PD having multiple facets in their journey. They also conveyed that there are limitations in the physiological activities, like immobility issues leading to reduced autonomy, which are associated with enormous non-motor complications impacting the dependence on a family member, caregiver, or support provider (P15). The disease condition results in severe cognitive impairment and speech problems, which are narrated as making comprehension and reasoning more difficult (P3).

Another older adult with PD has reported that there is a lack of orientation in the supportive environment, which emerges out of memory issues, but in turn acts as a coping strategy. They also reported feelings of embarrassment, loss of self-confidence, and difficulties related to cognitive adjustments. Older adults with Parkinson’s disease usually rely on manual reminders to perform the daily routine (P13).

“Even lacing my shoes has turned into a chore. It is very hard to tackle all these symptoms” (P15).

“Sometimes I forget the names of individuals I’ve known for years. It’s humiliating” (P3).

“I find myself forgetting things, which irritates me a lot” (P13).

### 3.4 Acclimatization to therapy

This theme presents how physical therapies aid older adults with PD in functioning independently, even though the motor symptoms cause severe pain, dizziness, vertigo, and low blood pressure. They elaborated on the efforts to continue therapy while living with PD. The physiotherapies provided can tailor the activities of everyday living (P10). Some of them expressed changes in their daily activities after therapy. Older adults with Parkinson’s disease continued struggling with symptom management (both cardinal and non-motor signs) while learning to cope with the disease (P7). They also reported that all the medicines, physiotherapeutic interventions, rehabilitation services, and maintenance expenditures have been taken care of by the homes they reside in or by their children. It is also stated that the consequences of therapies and medications are challenging and disruptive when they are overloaded with comorbid conditions. Intake of medicine for both Parkinson’s disease and pertinent comorbidities might be a tiring task for them (P14).

“Although physiotherapy is challenging, it keeps me moving. It enables me to cope with the disease” (P10).

“Tremors subside, but nausea is hard to bear, I say with a sense of challenge. Sometimes it happens consecutively, like this” (P7).

“These tablets do help me a great deal in managing my condition, but it is making me feel nauseated at times. At that time, if the caregiver is not here, it will be even worse, leading to a very unpleasant situation” (P14).

### 3.5 Need for a support network

This theme relates to older adults with PD longing for care and affection from their family members and close relatives. The participants conveyed that long-term care facilities deliver crucial assistance, therapeutic measures, and emergency support. Nevertheless, older adults with Parkinson’s disease expressed a desire for affection, care, and support. The significance of affection, emotional support, and companionship was emphasized by one of the participants (P13). They also reported that their children take up their role, and this shift in role makes them feel guilty, since their inability to be helpful in simple chores (P8). They acknowledged the efforts taken by the caregivers and feel grateful. One of the participants expressed feelings of guilt and gratitude toward their caregivers (P4). Some also described receiving companionship and support from peers in long-term care facilities.

While most emphasized the importance of family support, one older adult with PD (P1) stressed the importance of peer support through support groups, stating that if the support group programs are organized often, then it will be much better for them to learn and execute the coping strategies and to be together. Social engagement, such as conversation with peer group members and fellow residents of the long-term care facilities, makes them feel alive (P1). But some participants experienced significant challenges in maintaining these vital connections. Despite their longing for relationships, neurofunctional limitations stopped them from engaging in social activities, wanting social connection while experiencing difficulties engaging in social activities. This quotation showcases their feelings of separation from loved ones and difficulties maintaining intimate relationships (P11).

They also reported that the fear of being judged, loneliness, and lowered social activities led to avoidance of being around or meeting their fellow peer group or their own family. They also feel socially isolated due to their movement disability and avoidance in their community (P7). “I do not need food, medication, injections, physical therapy, exercises, or any of the other needs; instead, I need the support of my family members. My son, daughter, grandchildren, and other relatives are around me for care, companionship, and affection, which has not been given to me since I came to this long-term care facility. I am left alone with no one to talk to or share my feelings.” (P13).

“My companion is now the main source of support; however, I feel guilty relying on her so much” (P8).

“My caregiver has assumed a very protective stance and comes to the senior living community a great deal, but I feel grateful” (P4).

“The Parkinson’s support group is like a second family to me” (P1).

“I shy away from social gatherings, sometimes even when my family members arrive, because I cannot keep up. Getting separated from the person we love is another form of extreme pain” (P11).

“I end up sitting on the porch, observing the world pass by. It’s as if I’ve become invisible” (P7).

### 3.6 Existential reflection

The theme represents how older adults’ reflective engagement with questions on their life meaning, purpose, and identity evolves over time. Unlike the immediate emotional distress, these narratives portray a longitudinal process of transformation with PD, from initial shock toward acceptance of reality and spiritual grounding. The older adults with PD shared about their understanding of the diagnosis and its interrelationship to their sense of self. This included finding a spiritual path through faith and prayer, reaching acceptance of the disease and redefining personal purpose and identity despite advancing functional decline. The older adults with PD explained their efforts to understand their diagnosis and relate it to their personal lives. They also narrated about both optimistic and pessimistic attitudes based on the subjective experiences from the diagnosis and the current state of the disability. They conveyed that they try to figure out new coping strategies to survive the life-threatening disease, such as faith grounding and indulging in religious practices that might enhance the quality of life (P6).

They conveyed that gaining insights into the nature of the disease and the way their circadian rhythm reacts to the aberrant signs. They narrated that they are seeking peace via personal reflection, relationships and faith (P9). Feeling gratitude toward the caregivers, family members, peers, support group members, therapists, physicians, and surgeons.

“My prayers made me to sustain with the strength I require to deal with each day” (P6).

“I have accepted that this disease is part of the journey of my life.” “But it is still a perpetual nightmare for me” (P9).

## 4 Discussion

The research utilized a social constructivist paradigm to interpret the subjective lives of older adults with PD recognizing that knowledge emerges from social interaction, cultural settings, and long-term care environments, rather than as objective universal experiences. The study comprehended the journey from diagnosis to ongoing treatment engagement. The qualitative data were gathered from 5 long-term care facilities, 4 of which were in the south zone and 1 in the north zone of Chennai, with institutional instead of locational factors determining outcomes.

Thematic analysis revealed six themes that chart a course of development, beginning with identity disruption, progressing through emotional and neurofunctional difficulties, shifting to therapeutic acclimatization and support requirements, and finally arriving at existential reflection and meaning-construction. Importantly, while the themes are ordered sequentially, the experience of participants often oscillates between themes, rather than progressing from one to another. Differences in marital status and demographic variables were related to differences in relational experience and perceptions of care, as evidenced in the subsequent thematic analysis.

### 4.1 Theme 1: identity and self-perception

The theme of self-identity and self-concept was in accordance with existing theories of chronic illness adjustment. These findings are consistent with biographical disruption theory, which argues that the difficulties associated with Parkinson’s disease disrupt the continuity of self because the pre-existing social and bodily identities are destabilized. This result corresponds with recent qualitative studies on the conflict between maintaining pre-illness identity and accommodating new bodily limitations among older adults (57).

#### 4.1.1 Demographic and cultural influences on identity

The initial stages of PD diagnosis provoke immediate psychological distress, compounded by age-related changes. Older adults (most 70–79 years) have identity concerns with PD symptoms intersected with emotional outbursts. The sample population is largely male (68.75%), reflecting gender differences that have been found in identity disruption. Males were challenged with traditional masculine ideals of bodily strength and autonomy, while female older adults with PD seemed to incorporate these constraints more easily into their pre-existing self-concept with minimally pronounced role-related distress (58). Recent diagnoses (0.6 months to 1 year) predict acute challenges with identity adaptation and attendant emotional turbulences. This combined analysis shows that the loss of identity is not a simple process but a complex one that takes place between the past and the present through demographic positioning.

### 4.2 Theme 2: emotional dysphoria

The emotional dysphoria theme uncovered the emotional experiences that exceeded conventional clinical definitions of depression in PD. Although previous studies have widely reported on depression prevalence within PD groups, the study delineated that the intricate emotional experience of fear of falling, concerns with social evaluation due to their inability, and feelings of helplessness are related to disease progression and functional limitations. The findings suggested that physiological and motor symptoms appeared to have an impact on the emotional responses. The older adults with PD’s descriptions of feeling “a burden” and having “betrayal of the body” were consistent with current understanding of illness narratives in which disease hinders the basic assumptions of bodily dependability (59). These affective experiences represent the immediate, acute psychological distress that varies from the subsequent adaptive existential processing described in Theme 6.

As indicated by the above-described pathway of development, existential processing is more of a later-stage adaptive process than a characteristic of immediate emotional dysphoria. This distinction highlights the importance of why existential reflection (Theme 6) is viewed as a later-stage adaptive process rather than a part of emotional dysphoria, thus refuting the idea of aging and late-stage PD being characterized by hopelessness and decline. The result thus indicates the presence of resilience based on the capacity for existential meaning-making among the older adults with this disease condition.

### 4.3 Theme 3: neurofunctional impact

The neurofunctional impact theme demonstrated the complex interplay between cognitive symptomatology and emotional well-being, confirming the recognition of PD as a multisystem disorder (24). Notably, some older adults deliberately chose not to engage with society to avoid the experience of being “unrecognized in a crowd,” which exemplifies how social attitudes toward disability contributed to the functional impact of neurological symptomatology. This scenario is also applicable to the relationship within proximity. Such perceptions may contribute to heightened psychosocial stress and challenges in social participation. Cognitive effort and memory difficulties corroborate existing research on cognitive impairment in PD (60). The cognitive symptoms played a mediating role in the mechanisms through which identity evolution (Theme 1) was socially enacted in everyday interactions.

#### 4.3.1 Demographic correlates of neurofunctional impact

Neurofunctional impacts illustrated cultural groundedness, with discrimination and prejudice toward older adults with PD reflected in participant narratives. High prevalence of walking difficulties (68.75%) directly undermined self-identity and independence. Vision problems (43.75%), speech difficulties (31.25%), and mobility challenges generated emotional dysphoria. Comorbidities, including diabetes (50%), hypertension (43.75%), and back pain (37.5%), compounded functional limitations. The stigma and fear of discrimination uncovered in this theme aligned with general disability perceptions, showing how societal attitudes toward disability intensify the functional consequences of neurological symptoms beyond medical pathology alone.

### 4.4 Theme 4: acclimatization to therapy

The term ‘Acclimatization to Therapy’ was deliberately chosen to encompass both psychological and physiological adjustments that older adults with PD may undergo frequently but gradually. This theme indexed the active involvement in adaptation processes, contrasting with traditional clinical frameworks. This result was compatible with the self-efficacy theory of Bandura (61) and Ellis et al.’s (2011) self-management research in PD (62). The Treatment compliance data unveiled the differential participation patterns: 93.75% medication compliance versus 56.3% to physiotherapy, indicating accessibility barriers or differences in perceived efficacy. Narratives of physiotherapy stated the independence, despite the occurrence of motor symptoms, supported evidence-based guidelines for the exercise interventions in PD (63).

#### 4.4.1 Treatment patterns and educational background

The varied treatment adherence potentially associates with the educational background. The high rate of medication compliance (93.75%) contradicted research that supported lower compliance in older adults with PD (64), possibly because of the structured medication management offered in long-term care facilities. The results are potentially a reflection of the structured medication administration that is done by the facility staff, thus overcoming barriers such as forgetfulness and medication burden that are prevalent in community-dwelling older adults. This relates to Maslow’s hierarchy theory. However, the hospital attendance patterns (31.25% rarely, 37.5% often) indicated variable therapeutic engagement. Limited formal education may have influenced health literacy and adherence outcomes. These patterns suggest that environmental structures within institutional settings facilitate therapy adaptation.

### 4.5 Theme 5: need for support network

The need for a support network theme highlighted the necessity of relational care as central to wellbeing, above technical medical interventions. Older adults with PD deliberately prioritized “support from family members” rather than “foods, medications, injections,” contradicting biomedical models prioritizing symptom control over psychosocial needs. This finding is in line with attachment theory application to chronic disease (65) and also supported person-centered care approaches in PD clinical guidelines (66). During the interviews, there were emotional outbursts that occurred together with the discussion on the support needs of families, indicating the high emotional value and importance of relational care. Role-reversal dynamics, with adult children taking on caregiver roles, align with gerontological research exploring intergenerational relationships (67). However, they also highlight PD-specific challenges, including unpredictable symptom fluctuation and ongoing functional decline.

#### 4.5.1 Social support dynamics and geographic distribution

The personal profile represents that married older adults with PD have adequate primary support. In contrast, the unmarried, separated, divorced, and widowed people thriving for social support. Gender differences emerged: Males resisted talking about emotional support or taking part in support activities, consistent with masculine values that devalue emotional expression, while female older adults with PD were more open to interaction with care staff and peer support activities, consistent with less cultural inhibition to assistance-seeking. Cultural potential impacts act as a deterrent to men seeking emotional support.

### 4.6 Theme 6: existential reflection

The theme of Existential Reflection encompasses the ability to build and generate meaning in the phase of progressive illness, consistent with theories of successful aging (68) and post-traumatic growth (69). While suffering from emotional distress, the participants experience the earlier stages of their experiences, and the process of existential reflection transforms into a significantly different procedure, which requires the older adults with PD to gain emotional maturity and mental stability. The narratives suggest an adaptive orientation toward both acceptance and engagement with life. However, achieving meaning-making requires serious cognitive processing mediated by processes that likely include selective vulnerability, helplessness, and acceptance of reality. Results showing that older adults with PD (80–89 years) processed more extensive existential crises were consistent with developmental theories referring to the meaning-making becoming a progressively more crucial task in later life (70). The function of religious practice and faith-based background seen in this study synchronized with Koenig et al.’s (2001) study of religion and health in older adults, indicating spiritual coping may act as a buffer against disease-related distress (71) in PD-specific settings.

#### 4.6.1 Age and existential processing

The mature age group and the burden of chronic diseases provided natural contexts for existential consideration, especially with the accumulation of functional disabilities. While, older adults in this study might be confronted with cognitive challenges often paired with dementia, Parkinsonism, and mild cognitive impairment. But, not all older adults with PD with mild cognitive impairment suffer from Dementia (only 20–50% of them suffer from Dementia) (73). The longer the diagnosis period and the presence of more comorbidities accelerated the existential inquiry. Those individuals who adapted successfully showed higher levels of acceptance of bodily limitations, contradicting deficit models of adaptation that focus solely on progressive decline and hopelessness in the late stages of Parkinson’s disease. The progression of the disease itself led people to either maladapt or adapt, with successful adaptation catalyzing the levels of bodily acceptance.

### 4.7 Integration and theoretical framework

The six themes together supported an overarching biopsychosocial model for understanding the lives of older adults with Parkinson’s disease, beyond traditional biomedical models. The developmental pathway from identity transition to functional recovery to existential reflection suggested specific interventions and support strategies that are stage-specific (72). Whereas the study adhered to the biopsychosocial model of illness, recognizing that disease affects individuals across socio-cultural, economic, psychological, and biological aspects (74, 75). Also, the results emphasized the social model of disability for neurological disorders and the way environmental barriers and social attitudes intensified functional impairments. The older adults with PD reports about discrimination and social exclusion underlined the necessity of more comprehensive societal interventions transcending mere individualistic medical interventions (76). Finally, the research offered evidence for PD populations’ resilience, capacity, specifically through the acclimatization to therapy and existential reflection themes. The resilience and adaptation theory perspective highlighted adaptive capacity over deficit models in chronic illness research (77).

### 4.8 Clinical implications

The models of clinical care should integrate the screening for identity evolution following diagnosis with PD, and referral systems for psychosocial services and maintenance of role functioning. It necessitates a paradigm shift from biomedical to person-centered care. Healthcare practitioners must acknowledge the older adults with PD as individuals with identity crises, rather than just people needing pharmacologic management. Clinical services should incorporate routine psychosocial screening for identity disruption following diagnosis, enabling early interventions that support role continuity and self-concept preservation. Generic treatments must be replaced with tailored interventions addressing PD-specific anxieties and existential distress. Family-based care must be systematically integrated into clinical practice, including the caregivers in care planning and psychosocial decision-making procedures. Holistic clinical approaches must simultaneously address emotional and existential demands alongside physical symptoms.

### 4.9 Policy implications

The policies for long-term care facilities must include structured approaches for family engagement, incorporating interaction opportunities and caregiver involvement in care planning. Person-centered approaches require sufficient staffing and mandatory social participation programs. Anti-stigma campaigns to direct attitudes toward neurological conditions must be given priority. Budgets must support interdisciplinary staff, including psychologists and social workers, with specific allocations for training family caregivers and community support. Also, policies for long-term care facilities must include the infrastructure and protocols for psychosocial support and family engagement. Without these policy changes, progress in symptom control alone will not be sufficient to address the overall psychosocial requirements of older adults with PD in long-term care facilities.

### 4.10 Contribution to literature

This research contributed to the existing literature by presenting an understanding of the long-term care context, which was less focused on earlier qualitative PD research. The Acclimatization to Therapy theme presented new insight into treatment adaptation strategies. The cultural barriers presented in the neurofunctional impact theme presented important additions to the understanding of social participation difficulty in PD.

This study establishes that psychosocial care addressing identity confusion and facilitating family connection must be prioritized equally with motor symptom management in older adults with PD in long-term care facilities. Healthcare providers must integrate biographical interruption screening and tailored psychological interventions into standard PD care protocols. Policymakers must mandate structural changes within long-term care facilities, promoting meaningful family engagement and peer support, not merely ensuring physical safety. The central imperative is person-centered care that addresses the whole human experience: emotional, social, and existential, alongside physical symptoms, which is not an enhancement to PD care but its fundamental requirement. Without this change, institutional care will continue to focus on symptom management, while ignoring the continuing isolation and depression documented during this study.

### 4.11 Limitations and future directions of the study

The findings have limited generalizability as experiences may vary based on disease stages and advancement. The research was carried out in an urban institutional healthcare setting. Therefore, the results are specific to the context and should be interpreted cautiously in terms of their applicability to other settings of care, like: rural long-term care, tertiary care hospitals, hospice or palliative care, tertiary care hospitals, and home-based care. The exclusion of residents with severe functional dependency may have resulted in an overrepresentation of relatively autonomous residents, thus influencing the proportion of experiences and perceptions reported. In addition, the inclusion of older adults with Parkinson’s disease (PD) up to stage 4 could restrict the understanding of experiences associated with stage 5 PD or young-onset PD. The cross-sectional design restricts interpretation to a particular point in time, while longitudinal studies allow tracking how experiences evolve with disease progression. The selection of a single culture and healthcare setting restricts analysis of more extensive patterns. While comparative studies may reveal a more comprehensive picture of the sociocultural impacts on older adults with PD.

## 5 Conclusion

This study highlighted the multifaceted challenges faced by patients with Parkinson’s disease in India. The findings suggested that identity evolution, emotional distress, social isolation, and motor dysfunctions are experienced with PD. Person-centered and culturally sensitive care providing, addressing all these dimensions simultaneously, is essential that expands beyond symptom control. Healthcare providers, policymakers, and caregivers must adopt inclusive methods that integrate family support, peer support, and coordinated interventions to enhance the overall quality of life. This study provided culturally oriented insights from South India into PD experiences and put forth evidence-based recommendations for improving geriatric care in the Indian situation. Future studies can explore these experiences in a wide range of regional contexts. Further research might investigate the longitudinal evolution of disease adaptation to provide a more comprehensive view of PD and inform the development of more responsive long-term care strategies.

## Data Availability

The original contributions presented in the study are included in the article/supplementary material, further inquiries can be directed to the corresponding author.
